# E-Clinical High Risk for Psychosis: Viewpoint on Potential of Digital Innovations for Preventive Psychiatry

**DOI:** 10.2196/14581

**Published:** 2019-10-03

**Authors:** Thomas Reilly, Andrea Mechelli, Philip McGuire, Paolo Fusar-Poli, Peter J Uhlhaas

**Affiliations:** 1 Department of Psychosis Studies Institute of Psychiatry, Psychology & Neuroscience King’s College London London United Kingdom; 2 Early Psychosis: Interventions and Clinical-Detection Lab Department of Psychosis Studies King’s College London London United Kingdom; 3 OASIS Service South London and Maudsley National Health Service Foundation Trust London United Kingdom; 4 Department of Brain and Behavioral Sciences University of Pavia Pavia Italy; 5 National Institute for Health Research Maudsley Biomedical Research Centre South London and Maudsley National Health Service Foundation Trust London United Kingdom; 6 Institute of Neuroscience and Psychology University of Glasgow Glasgow United Kingdom

**Keywords:** psychotic disorders, schizophrenia, prognosis, treatment, clinical high risk, digital, e-health, internet, smartphone, mobile phone

## Abstract

E-mental health is an emerging area of research that has the potential to overcome some of the current barriers to progress in working with people at clinical high risk for psychosis (CHR-P). This article provides an overview of how e-mental health could be used in the detection, prediction, and treatment in the CHR-P population. Specifically, we evaluate e-detection, e-prediction, and e-therapeutics for this clinical population. E-mental health holds great promise to improve current management of CHR-P individuals.

## Introduction

The identification of people at clinical high risk for psychosis (CHR-P) [1] offers a unique opportunity to alter the illness course of psychotic disorders [2]. These individuals often have several risk factors for psychosis [3] and typically present with attenuated psychotic symptoms in the context of a recent decline in functioning [4]. They display symptoms and functional impairments that are qualitatively similar to those observed in established mental disorders [5]. The risk of transition to psychosis within 2 years in these individuals is approximately 20% [6]. This risk is not the same across the different CHR-P subgroups. In particular, individuals meeting criteria for short-lived psychotic episode show a distinctive and very high risk of developing persistent psychotic disorders that cumulates to about 50% at 2 years [7]. CHR-P individuals presenting with a short-lived psychotic episode that is spontaneously remitting but characterized by disorganized behavior have an even higher risk of developing a persistent psychotic disorder, cumulating at 89% at 5 years [8]. These individuals also have unmet clinical needs not typically addressed by the current configuration of mental health services [9,10]. Overall, the risk for the development of psychosis from a CHR-P stage has declined from 29% [11] to 20% [6] in recent years, although not across all sites [12]. This variable transition risk is due to different sampling strategies being adopted to recruit these individuals [13]. Emerging evidence indicates that the method of recruitment prior to a CHR-P assessment is fundamental in enriching their actual level of risk for psychosis [13].

**Figure 1:**
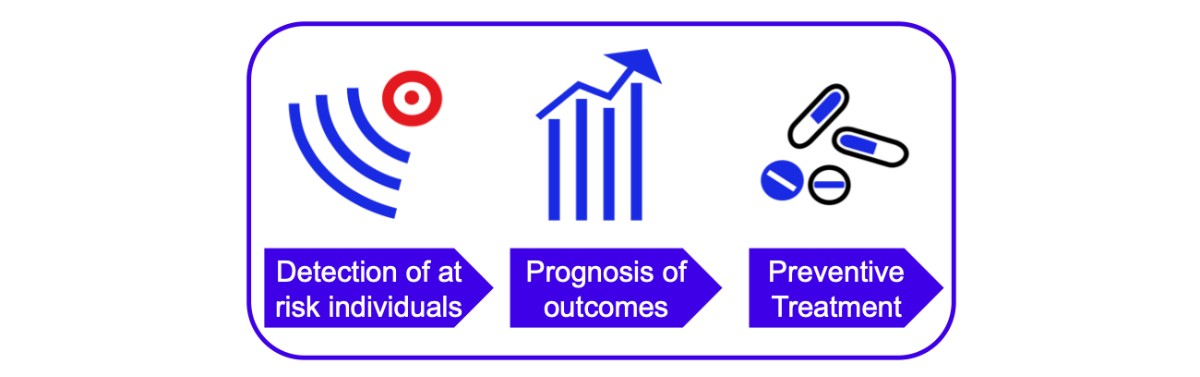
Core clinical and research components for effective prevention of psychosis, from Fusar-Poli et al [35].

Although the CHR-P paradigm has been adopted in various countries worldwide [14-17], a number of challenges have arisen, hindering its penetrance into mainstream clinical practice. The real-world success of the CHR-P paradigm rests on three core components: an efficient detection of at risk cases, accurate prediction of their outcomes, and effective preventative treatments to alter the course of these outcomes Figure 1.

There are currently barriers to the implementation of each component. First, well-established CHR-P services [17] detect as low as 5% of first episode psychosis (FEP) patients before illness onset [18]. Even within national youth mental health services, the proportion of FEP cases detected at their CHR-P stage is only 12% [19]. Second, although the prognostic performance of the current CHR-P psychometric tools is excellent [20], it is highly dependent on the way CHR-P individuals are recruited [21]. Furthermore, prognostic outcomes in this population are mostly based on group-level predictions, and it is not yet possible to forecast the onset of psychosis at the individual-subject level [22]. This is a substantial limitation given that the CHR-P group is highly heterogenous and includes different subpopulations with differing outcomes [7]. Third, no specific preventive treatment appears to be more effective than others in preventing onset of psychosis [23], treating attenuated positive symptoms [24,25], treating attenuated negative symptoms [26], treating depressive symptoms [27], reducing distress [28], or improving social functioning [29]. Again, the lack of evidence may partially reflect the fact that one-size-fits-all approaches for this population do not work and individualized treatments should be offered instead.

E-Mental health is a new approach that may be uniquely suited to overcome some of these barriers. Individuals at CHR-P have a young age range (most criteria are set at 14 to 35 years) and as such tend to be highly engaged with the digital world [30]. Although previous reviews have highlighted the emerging potential for e-mental health in psychosis [31-34], there is currently no overview focusing on the potential and prospects specifically in CHR-P. In this article, we provide an overview how e-mental health can be applied to the detection, prediction, and treatment of CHR-P. Studies included in this overview are summarized in Table 1. Although this is not a systematic review and there is no assumption that the literature surveyed is comprehensive, we provide our search strategy and inclusion criteria in Multimedia Appendix 1.

## E-Detection

Detecting individuals at risk prior to the onset of psychosis has been a key research priority over the past two decades since the Personal Assessment and Crisis Evaluation study first developed the concept of the CHR-P [16]. Typical inclusion in the CHR-P group is based on attenuated psychotic symptoms, brief episodes of psychosis, or functional deterioration in those with genetic vulnerability for psychosis [35,36]. A distinct approach to the identification of CHR-P individuals is the basic symptom concept proposed by Huber and colleagues [37]. Basic symptoms involve self-experienced perceptual and cognitive anomalies that are thought to represent the earliest manifestation of psychosis risk [38]. More recent studies have shown that the combined presence of both basic symptom and CHR-P criteria increases the predictive power significantly [39]. However, it should also be noted that CHR-P participants who do not make the transition to psychosis are characterized by extensive psychiatric comorbidity and reduced occupational and social functioning [40]. Accordingly, these two domains are also potentially important for targets for e-mental health applications, in terms of both prediction and preventive treatments.

Recruitment strategies have a significant impact on subsequent transition rates of CHR-P cohorts, with self-referrals, assertive community outreach, and population screening associated with lower rates of transition to psychosis [41]. Despite the expansion of CHR-P services, only a small proportion of FEP patients are detected [42]. It is thus a key priority for CHR-P to improve detection of individuals prior to the FEP while not diluting the sample’s baseline risk of psychosis. As the majority of FEP patients actively seek information regarding mental health issues online as their symptoms first develop [43], early identification of CHR-P individuals may be possible through digital detection strategies. These could include online screening as well as use of social media information. Recent evidence, for example, suggests that references to sadness, loneliness, hostility, rumination, and increased self-reference on Facebook predict later onset of depression [44]. A similar approach may be useful in CHR-P as the development of psychosis is characterized by linguistic anomalies that can be detect by automated speech analysis [45,46].

Online screening has the potential to reach a greater number of individuals compared with traditional routes of referral to CHR-P services. McDonald et al [47] used a website for detection of CHR-P in the community. Potential participants were invited via email, flyers, and posters to a website [48] and then asked to complete the 16-item version of the prodromal questionnaire (PQ-16) and 9-item questionnaire of perceptual and cognitive aberrations (PCA) for basic symptoms. This allowed screening of a large number of individuals, 52.3% (1202/2296) of whom met PQ-16 cutoff criteria and 73.6% (1691/2296) of whom met PCA cutoff criteria. Of those meeting screening cutoff criteria who then attended a clinic interview, 31.2% (101/324) met clinical CHR-P criteria. Importantly, a subset of 8 individuals (2.5%) also met criteria for FEP. Receiver operating characteristic curve analysis revealed good to moderate sensitivity and specificity for predicting CHR-P status based on the online results [47]. A machine-learning approach that selected all 25 items from both the PQ-16 and the PCA in addition to demographic variables lead to an improved specificity of 57% while only marginally affecting sensitivity (81%), compared with the original online screening tool.

This study suggests that online screening of community samples for emerging psychosis is possible, potentially identifying a large number of people meeting CHR-P criteria. However, it is currently unclear how many of these participants meeting CHR-P criteria will actually develop psychosis. Accordingly, 2-year transition rates are needed to validate whether the sample detected are truly at risk of developing a psychotic disorder or whether they are false positives. A similar approach to McDonald et al [47] has been implemented using the 32-item self-screen prodrome questionnaire [49], although, again, long-term transition rates are not known. Furthermore, the validity of self-screening questionnaires, particularly when conducted in nonclinical populations, has been questioned [50] due to poor prognostic performance in predicting subsequent psychosis.

More targeted screening of populations accessing secondary mental health services that use electronic health records is also possible through online screening. Our group developed a psychosis risk calculator for patients already accessing secondary mental health services [18]. This tool uses routine clinical data including *International Classification of Diseases, Tenth Revision* (ICD-10), spectra diagnoses to predict future risk of developing a psychotic disorder and has been externally validated twice in different National Health Service (NHS) Trusts [51] showing an acceptable prognostic performance (Harrell C of 0.73). Patients who have accessed secondary mental health services have a 5-fold increased risk compared with the general population [18], suggesting this may be an efficient way of detecting new cases of psychosis while not diluting the level of risk in the sample. As it uses routine clinical data, the calculator could be used to automatically screen electronic health records for those at increased risk of future psychosis. However, it does rely on the assessment of health professionals to provide ICD-10 diagnoses, and therefore it cannot be used universally for self-screening. The calculator is one of the few eHealth tools in the CHR-P population that is being implemented into clinical routine practice [52].

The North American Prodrome Longitudinal Study has produced an online risk calculator for individuals meeting CHR-P criteria [53]. It showed an overall accuracy of 72% in predicting psychosis when validated in an independent external data set [54]. However, it requires input in the form of a structured interview to confirm CHR-P status and neuropsychological testing, limiting its applicability to wider clinical populations.

E-Mental health offers the opportunity for efficient, scalable screening of those at risk of psychosis across populations that are currently not reached by conventional recruitment, potentially allowing better detection of individuals prior to their FEP. Preliminary evidence suggests that it is indeed feasible. The next step is determining whether these tools can be implemented in practice to identify individuals at increased risk of psychosis while not diluting the overall risk of the sample with false positives.

## E-Prognosis

The risk of transition to psychosis from CHR-P is maximal within the first 2 years [55]. This period is therefore a crucial time for predicting onset of psychosis. Traditionally, this has been achieved through regular clinical monitoring from CHR-P services to assess for transition to psychosis, but advances in mobile phone technology offer the opportunity for a far greater temporal resolution in monitoring changes in mental state that may occur on a daily or even momentary basis.

The experience sampling method (ESM) uses mobile phones to measure self-rated changes in mental state on a daily basis [56]. It has excellent ecological validity and allows close monitoring of mental state, particularly in regard to predictors of transition to psychosis. ESM techniques in CHR-P were initially rudimentary, requiring participants to fill out responses in a paper diary when prompted by a wristwatch alarm [57,58]. Palmier et al [59] demonstrated that it is feasible to monitor symptoms through a mobile phone app in a small sample (n=12) of CHR-P individuals, paving the way for further cross-sectional ESM studies. Klippel et al [60] showed in 46 CHR-P individuals that momentary stress increased psychotic experiences via affective disturbance using the PsyMate app. Reininghaus et al [61] used ESM to link threat perception to psychotic experiences in 44 CHR-P individuals and in another study [62] to show an association with sensitivity to outsider status and aberrantly salient experiences with psychotic experiences. Van der Steen et al [63] used ESM to demonstrate an association between affective and psychotic experiences in response to stress in CHR-P individuals, showing an association between stress and psychotic experiences. ESM has shown potential utility in monitoring fine-grained changes in psychopathology during the development of psychosis; however, longitudinal studies are needed to determine its significance for predicting the onset of psychosis. To date, one longitudinal study has been registered: Booij et al [64] plan to predict outcome in various stages of psychosis using mobile phone diary measures of symptoms, stress, emotions, and functioning.

Digital phenotyping, also known as personal sensing, is a novel investigational technique whereby passive measures of mobile phone activity are recorded in real-time [65], which has been postulated to provide a digital phenotype of psychiatric disorders [66]. These measures may include the participant’s interaction with their phone (eg, call logs, number of messages, keyboard use) as well as measures of their activity and movement (eg, through accelerometers or Global Positioning System tracking). Passive data provide a continuous readout every day and require no active role by the participant, thus providing significant advantages over traditional, episodic cross-sectional data. Passive data are also potentially more ecologically valid than symptom ratings elicited by standard questionnaires or interviews, and there is reduced risk of subject attrition.

However, the use of passive mobile phone measures warrants some important ethical considerations [65]. As with any investigation, informed consent, data security, and anonymization are crucial. Additionally, in those with psychotic disorders, care must be taken not to exacerbate paranoia when using participants’ personal devices. Passive measures therefore may be particularly suited to CHR-P individuals, who retain insight, particularly for the prediction of psychosis onset. Unlike ESM, they do not require continual user input and may therefore be less burdensome to participants. A pilot study suggests digital phenotyping could predict relapse in schizophrenia based on anomalies in patient behavior [67]; further studies in CHR-P populations are underway, for example, using the MindStrong app [68].

ESM and digital phenotypes have complementary strengths. ESM provides explicit information about a patient’s state of mind and as such is key to understanding their motivations and behaviors, whereas digital phenotyping has the important advantage of placing minimal burden on the patient apart from keeping their mobile phone charged and as such is ideal for clinical translation. Initial studies suggest that it might be possible to use passive data generated via digital phenotyping as a proxy for active data collected via ESM [69]; in the context of CHR-P, this could enable the background monitoring of risk of transition to psychosis with minimal interference on the day-to-day life of patients.

**Figure 2:**
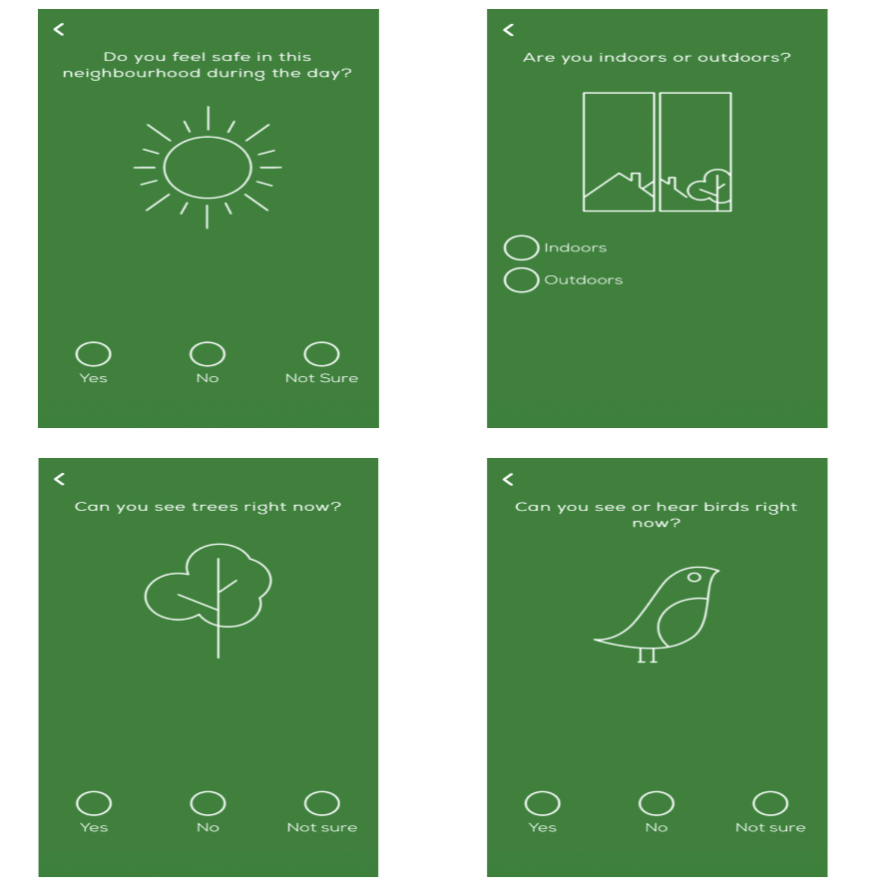
Urban Mind app user interfaces.

In addition to informing diagnosis, prognosis, and treatment, e-tools could be used to gain greater understanding of the mechanisms that underlie transition to psychosis. For example, our research team is currently using the Urban Mind app [70] to investigate the impact of the surrounding social and physical environment on risk of transition to psychosis in individuals at CHR-P (see Figure 2 for user interface).

These studies suggest that e-mental health has potential value in identifying risk factors for subsequent transition to psychosis and predicting the onset of psychosis. In addition, these approaches may also be applied toward prognosis for outcomes other than transition to psychosis, such as functional outcomes and development of nonpsychotic disorders. ESM and digital phenotyping are two appealing methodologies with great promise and are ideally suited to longitudinal designs to examine the development of clinical trajectories in CHR-P populations.

## E-Treatment

One criticism of CHR construct is that there is no treatment conclusively shown to prevent the onset of psychosis [71]. However, there are several promising therapeutic strategies currently under investigation that could significantly reduce psychosis risk and address important additional areas of impairment—in particular, cognitive deficits and social and occupational functioning.

Since individuals at CHR-P do not have a mental disorder as such, any treatment has to be well tolerated and acceptable. Moreover, by definition these individuals retain a degree of insight into their symptoms [72], offering a significant therapeutic window of opportunity prior to the onset of psychosis. Guidelines currently suggest psychological therapy and recommend not treating with antipsychotic medication [73]. As various psychological therapies can be delivered online, e-mental health has the potential to enhance access to treatments and reduce costs. A systematic review and meta-analysis suggested that guided online cognitive behavioral therapy (CBT) for various psychiatric disorders had equivalent effect sizes compared with face-to-face interventions [74], highlighting the potential utility of administering CBT through digital modalities. Other psychological interventions such as mindfulness, which has shown some efficacy for psychosis [75], may also be effective when provided online [76]. Alvarez-Jiminez et al [77] conducted a novel strengths and mindfulness-based online social therapy for individuals at CHR-P. Participants found the intervention acceptable and showed an improvement in social functioning and subjective well-being, warranting further study.

Digital interventions showing promise in established psychosis, relevant to CHR-P, include avatar therapy [78], online CBT skills program for hallucinations [79], mobile phone apps delivering therapeutic interventions [80-82], and online peer-to-to peer support networks, reported to increase feelings of social connectedness [83]. Indeed, Rice et al [84] have developed an e-mental health service providing clinician-delivered Web chat counseling, a moderated peer-to-peer social network, and user directed online therapy. This enhanced moderated online social therapy is seeded in eheadspace, part of headspace, a flagship Australian youth mental health program with an integrated early intervention in psychosis component [85].

An important aspect of the clinical presentation of CHR-P is cognitive deficits across a range of domains associated with transition to psychosis [86,87]. One way of targeting cognitive deficits in CHR participants could be cognitive remediation (CR)–based treatments [88] that can be administered via computerized training procedures [89]. These approaches improve neural circuits underlying cognitive deficits with significant impact on social functioning [90], especially if administered in the early stages of schizophrenia [91]. There is emerging evidence that CR treatments may be effective in targeting cognitive dysfunctions in CHR participants. Loewy et al [92] examined the effects of CR administered through laptop and home computers on verbal learning and memory. CR significantly improved verbal memory (effect size = 0.61) as well as positive symptoms. Hooker et al [93] presented a pilot uncontrolled study of cognitive training delivered online for CHR-P, showing this intervention is feasible and appears to provide improvements in the global cognition in response to training.

Digital interventions could also be extended to address functional impairments in CHR-P individuals. In a recent study, Schlosser et al [82] examined a mobile-based digital health intervention designed to improve motivation and quality of life in young people with schizophrenia. Compared to the control group, the active treatment arm demonstrated significant improvements in levels of depression, defeatist beliefs, and self-efficacy as well as a trend for improved negative symptoms. These improvements were maintained 3 months after the end of trial. Accordingly, these data suggest that mobile-based interventions could be useful for addressing important domains of functioning in CHR-P populations.

Virtual reality (VR) is an emerging tool for the treatment of a wide range of mental disorders that involves interactive computer-generated worlds in which therapeutic strategies can be implemented and tested [94]. In established schizophrenia, VR approaches have shown preliminary efficacy in targeting positive symptoms, such as delusions [95] and hallucinations [96], as well as increasing social participation [97]. VR has also shown potential as a treatment adjunct in psychosis in modalities such as cognitive remediation and social skills training [98], showing preliminary promising results.

Following successful pilot research demonstrating the safety of VR in the CHR-P population [99], this technique has been used to investigate the effect of various simulated social environments on psychopathology. A simulation of the London underground transport system has been used to investigate paranoid ideation [100,101] and perceived ethnic discrimination [102], while café [103] and bar simulations [104] have been used to investigate the effect of social stress on interpersonal distance and paranoid ideation, respectively. These studies show the possibilities of using VR to study psychopathology in a simulated environment. Arguably, VR may be better suited as a treatment modality in CHR-P compared with more established stages of psychosis.

**Table 1:** Summary of included studies of e-mental health in clinical high risk for psychosis.

Study and type		Summary	Results
**E-Detection**			
	McDonald et al 2018 [47]	Web-based community screening for CHR-P^a^.	Good to moderate sensitivity and specificity for predicting CHR status based on online screening.
	Fusar-Poli et al 2017 [18]	Risk calculator based on routine clinical data of patients accessing secondary health services.	Acceptable predictive performance; Harrell C of 0.80 (0.79-0.82).
	Fusar-Poli et al 2018 [51]	External validation of the above study in a second NHS^b^ trust.	Acceptable predictive performance; Harrell C of 0.73.
	Fusar-Poli et al 2019 [52]	Protocol for implementation study of the above into routine clinical care.	N/A.
	Cannon et al 2016 [53]	Risk calculator based on specialized clinical assessment.	Acceptable predictive performance; Harrell C of 0.71.
	Carrión et al 2016 [54]	External valiation of the above study in a second cohort.	Good discrimination area under the curve of 0.790 (0.644-0.937).
**E-Prognosis**			
	Palmier-Claus et al 2012 [59]	Feasibility of smartphone self-report of symptoms using the ClinTouch app.	A total of 82% (36/42) of participants were compliant with the smartphone measures.
	Klippel et al 2017 [60]	ESM^c^ study of stress and psychotic symptoms using the PsyMate app.	Effects of stress on psychotic experiences were mediated through affective disturbance.
	Reininghaus et al 2016 [61]	ESM study of threat perception and psychotic experiences using PsyMate app.	Outsider status and threat anticipation were associated with more intensive psychotic experiences in those who experienced sexual abuse compared with those exposed to low levels of sexual abuse.
	Reininghaus et al 2016 [62]	ESM study of sensitivity to outsider status, salient experiences, and psychotic experiences using the PsyMate app.	Elevated stress sensitivity, aberrant salience, and enhanced threat anticipation were associated with increased intensity of psychotic experiences.
	van der Steen et al 2017 [63]	ESM study of affective and psychotic experiences in response to stress using a digital wrist watch to instruct participants to enter written self-report at random time points.	Greater associations between negative affect and stress compared with psychotic patients (*P*=.008) and controls (*P*<.001).
**E-Treatment**			
	Alvarez-Jimenez et al 2018 [77]	Pilot study of the online social therapy intervention, Momentum.	Of the 70% actively engaged during the study, all reported positive experiences, considered it safe, and would recommend it to others; 93% reported it to be helpful. Large improvements in social functioning (*d*=1.83, *P*<.001) and subjective well-being (*d*=0.75, *P*=.03).
	Rice et al 2018 [84]	Study protocol for enhanced moderated online social therapy (MOST).	N/A.
	Loewy et al 2019 [92]	Randomized trial investigating the effectiveness of auditory-processing exercises administered through laptops in CHR-participants compared to the effects of computer games (CG) training.	Targeted cognitive training showed a significant improvement in verbal memory compared to CG participants (effect size = 0.61). Positive and total symptoms improved in both groups over time.
	Hooker et al 2014 [93]	Pilot uncontrolled study of online cognitive training.	Significant improvements in processing speed (*P*=.01, *d*=0.63) and nonsignificant improvements in visual learning and memory (*P*=.06, *d*=0.54) and global cognition (*P*=.06, *d*=0.45).
	Valmaggia et al 2007 [99]	Study of the feasibility and safety of VR^e^ environments.	No adverse events; no increase in mean anxiety score (*P*=.29).
	Valmaggia et al 2015 [101]	VR study assessing childhood bullying and paranoid ideation in a simulation of the London underground.	More paranoid appraisals of VR simulations compared with controls (*P*<.001).
	Valmaggia et al 2015 [100]	VR study assessing social defeat and paranoid appraisals in a simulation of the London underground.	More paranoid ideation during VR simulation compared with controls χ2(1)=21.06, (*P*<.001).
	Shaikh et al 2016 [102]	VR study assessing ethnic discrimination and persecutory paranoia in a simulation of the London underground.	Higher levels of perceived ethnic discrimination correlated with greater paranoid persecutory ideation in VR environment r=0.25, *P*=.009.
	Geraets et al 2018 [103]	VR study assessing interpersonal distance regulation in a simulated café.	Interpersonal distance increased when social stressors were present in the environment F=3.02, *P*=.02.
	Veling et al 2016 [104]	VR study assessing paranoia in a simulated bar environment.	Increased paranoia compared with controls, regression coefficient 3.80 (95%CI 0.24–7.37) *P*=.04.

^a^CHR-P: clinical high risk of psychosis.

^b^NHS: National Health Service.

^c^ESM: experience sampling method.

^d^VR: virtual reality.

## Conclusion

Digital technologies are at present underused as a research or clinical tool for CHR-P and may be ideally placed to address the current challenges of the field. These include detecting those at risk of psychosis outside specialized CHR-P clinics, monitoring to predict future development of psychosis, identifying digital biomarkers for psychosis and other clinical outcomes, and delivering novel treatment modalities. Furthermore, individuals at CHR-P, by definition young and help-seeking, are ideally suited to digital interventions. However, longitudinal studies of digital technologies are required to assess which measures are useful for predicting risk of psychosis in individuals identified by online screening. Future research is required to rigorously test whether digital interventions have a place in the detection or management in people at CHR-P. In addition, we need greater understanding of the relationship between the different types of measures (eg, active and passive data collected via ESM and digital phenotyping, respectively) in order to develop and validate e-tools that provide maximal information while minimizing burden on the patient.

## Appendix

Multimedia Appendix 1 Search strategy and inclusion criteria.

## Abbreviations

CBT: cognitive behavioral therapy
CHR-P: clinical high risk for psychosis
CR: cognitive remediation
ESM: experience sampling method
FEP: first episode psychosis
ICD-10: International Classification of Diseases, Tenth Revision
NHS: National Health Service
NIHR: National Institute for Health Research
PCA: 9-item perceptive and cognitive aberrations questionnaire
PQ-16: 16-item prodromal questionnaire
VR: virtual reality
