# Supplementary material for: E-Clinical High Risk for Psychosis: Viewpoint on Potential of Digital Innovations for Preventive Psychiatry
Source: JMIR Ment Health. 2019 Oct 3;6(10):e14581. doi: 10.2196/14581 (PMC6915798; doi:10.2196/14581)
Supplement: Multimedia Appendix 1 [file mental_v6i10e14581_app1.pdf]

SUPPLEMENTARY MATERIAL

Search Strategy

PubMed was searched from inception to February 2019. The following combinations of search terms were used:

(internet OR online OR app OR smartphone OR mobile OR smartphone OR e-mental health OR e mental health OR digital OR mobile OR ESM OR experience sampling OR GPS OR accelerometer OR microphone OR global positioning system)

AND (psychosis AND (CHR OR UHR OR ARMS OR risk)

Further relevant publications were identified through the expert authors' own literature collection.

Inclusion Criteria

We included research published in peer review journals. We included studies of CHR-P individuals defined by standard psychometric instruments: Comprehensive Assessment of At Risk Mental States, Brief Psychiatric Rating Scale, Structured Interview for Psychosis-risk Syndromes, Basel Screening Instrument for Psychosis. We included studies investigating any aspect of 'E-Mental Health'; for example, online screening, smartphone research, virtual reality research.
